# PD‐L1 blockade by immune checkpoint inhibitors impairs sensitivity to osimertinib in EGFR‐mutant non‐small cell lung cancer cells

**DOI:** 10.1002/cai2.39

**Published:** 2022-12-07

**Authors:** Dantong Sun, Puyuan Xing, Junling Li

**Affiliations:** ^1^ National Cancer Center/National Clinical Research Center for Cancer/Cancer Hospital, Chinese Academy of Medical Sciences and Peking Union Medical College Beijing China

**Keywords:** combination therapy, durvalumab, osimertinib

## Abstract

In the previous studies, it was shown that osimertinib plus durvalumab did not achieve satisfactory efficacy, even inferior to osimertinib alone. We found that PD‐L1 blockade impaired the efficacy of osimertinib in EGFR‐mutant NSCLC cells despite the presence of the tumor microenvironment. Therefore, durvalumab has no synergistic effect on osimertinib, and combination therapy will not enhance the efficacy of osimertinib.

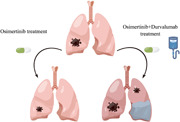

**Abbreviations**
EGFR: epidermal growth factor receptor
ICIs: immune checkpoint inhibitors
ILD: interstitial lung disease
NSCLC: non‐small cell lung cancer
ORR: objective response rate
PD‐L1: programmed cell death ligand 1
TKI: tyrosine kinase inhibitor

In recent years, the management of advanced non‐small cell lung cancer (NSCLC) with epidermal growth factor receptor (EGFR) mutation has developed rapidly. Osimertinib is a third generation EGFR tyrosine kinase inhibitor (TKI), which has achieved satisfactory efficacy and tolerability as the first‐line (FLARUA study [ref]) and second‐line (AURA series of studies [ref]) treatment of patients with EGFR‐mutant NSCLC. This suggested that EGFR signaling is involved in the upregulation of programmed cell death ligand 1 (PD‐L1) expression, which induces apoptosis of T cells, thereby promoting immune escape of tumor cells [1]. Therefore, several studies were conducted to explore the efficacy and safety of osimertinib combined with PD‐L1 monoclonal antibody durvalumab in patients with EGFR‐mutant NSCLC. The TATTON study is a phase Ib trial of osimertinib combination therapy, including durvalumab, in treatment of advanced EGFR‐mutant NSCLC. Results showed that the combination of osimertinib and durvalumab resulted in an objective response rate (ORR) of 43%, but an increased incidence of interstitial lung disease (ILD) (22%). Another phase III study of osimertinib versus osimertinib plus durvalumab, the CAURAL trial, whose recruitment was terminated early due to the high incidence of ILD in the TATTON trial, showed an ORR of 80% in the osimertinib arm compared with an ORR of 64% in the combination arm [2]. Therefore, the combination of osimertinib plus durvalumab did not appear to provide a significant improvement in efficacy compared with osimertinib alone [3]. Regardless of the impact of durvalumab on the tumor microenvironment and the high incidences of adverse events, whether PD‐L1 blockade itself has a synergistic effect on osimertinib treatment is still unknown. The role of PD‐L1 blockade in the treatment of EGFR‐mutant NSCLC with osimertinib, regardless of the effect on the tumor immune microenvironment, has not been reported. Here, we used a human EGFR‐mutant NSCLC cell line HCC4006 (ATCC® CRL‐2871™) to test the IC50 of osimertinib alone or in combination with different concentrations of durvalumab to explore the effect of osimertinib in blocking PD‐L1.

After seeding cells in 96‐well plates, the cells were treated with a concentration gradient of osimertinib (manufacturer, city and country) ranging from 1 to 40 µm, diluted in serum‐free medium alone or with 0.5 mg/mL durvalumab (manufacturer, city and country), 1 mg/mL durvalumab or 2 mg/mL durvalumab, respectively. After an additional 24 h of incubation, an MTS assay was performed to examine the IC50 of osimertinib against EGFR‐mutant NSCLC cells using different solvents. It was proved that the IC50 of osimertinib continued to increase with the increase of durvalumab concentration compared with osimertinib alone, which was 6.202 µm, 9.28 µm, 13.37 µm, and 15.18 µm for osimertinib plus 0.5 mg/mL durvalumab, osimertinib plus 1 mg/mL durvalumab and osimertinib plus 2 mg/mL durvalumab, respectively (Figure 1). Currently, there is still a lack of evidence for first‐line osimertinib combined with durvalumab or other immune checkpoint inhibitors (ICIs) in patients with EGFR‐mutant NSCLC. In the above‐mentioned previous studies, it was shown that combination therapy did not achieve satisfactory efficacy, even inferior to osimertinib alone. Durvalumab blocks PD‐L1 on the surface of tumor cells and relieves the suppressive effect of immune cells in the tumor stroma. However, based on in vitro experiments, we found that PD‐L1 blockade impaired the efficacy of osimertinib in EGFR‐mutant NSCLC cells despite the presence of the tumor microenvironment. Therefore, durvalumab has no synergistic effect on osimertinib, and combination therapy will not enhance the efficacy of osimertinib. In summary, we should be cautious when choosing first‐line EGFR‐TKIs combined with immunotherapy.

**Figure 1:**
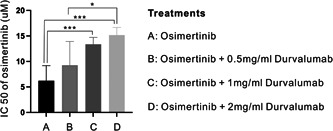
The IC50 of osimertinib in EGFR‐mutant NSCLC cells, diluted in serum‐free medium alone, or combined with 0.5 mg/mL durvalumab, 1 mg/mL durvalumab or 2 mg/mL durvalumab, respectively. EGFR, epidermal growth factor receptor; NSCLC, non‐small cell lung cancer.

## AUTHOR CONTRIBUTIONS

*Conception/design*: Dantong Sun and Junling Li. *Collection and/or assembly of data*: Dantong Sun and Puyuan Xing. *Manuscript writing*: Dantong Sun and Junling Li. *Final approval of manuscript*: All authors.

## CONFLICT OF INTEREST

The authors declare no conflict of interest.

## ETHICS STATEMENT

All experiments were carried out following the National Health and Family Planning Commission of the Professional Regulation Commission (PRC) guidelines.

## INFORMED CONSENT

Not applicable.

## Data Availability

Data supporting this report are available from the corresponding authors on reasonable request.
